# Supplementary material for: A One-Pot Convenient RPA-CRISPR-Based Assay for Salmonella enterica Serovar Indiana Detection
Source: Microorganisms. 2024 Mar 5;12(3):519. doi: 10.3390/microorganisms12030519 (PMC10974848; doi:10.3390/microorganisms12030519)
Supplement: Supplementary file 1 [file microorganisms-12-00519-s001.zip › microorganisms-2871793-supplementary.pdf]

Supplementary files

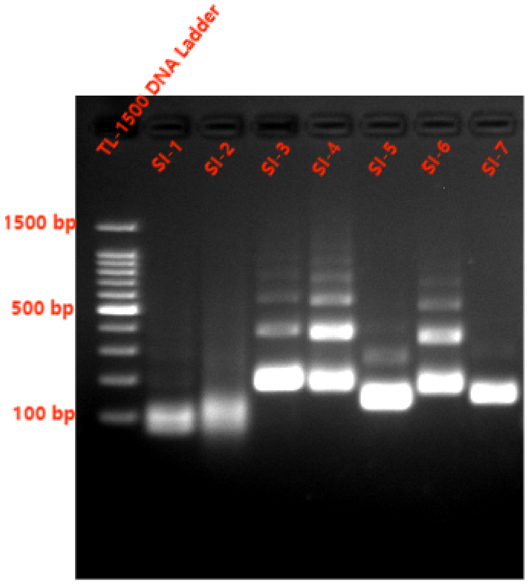

Supplementary Figure S1. The amplification products of RPA assay by using 7 pair's primers.
